# Development of Nanoemulsions to Enhance the Antileishmanial Activity of* Copaifera paupera* Oleoresins

**DOI:** 10.1155/2018/9781724

**Published:** 2018-04-04

**Authors:** Igor A. Rodrigues, Aline de S. Ramos, Deborah Q. Falcão, José Luiz P. Ferreira, Silvia L. Basso, Jefferson Rocha de A. Silva, Ana Claudia F. Amaral

**Affiliations:** ^1^Department of Natural Products and Food, School of Pharmacy, Federal University of Rio de Janeiro, 21941-590 Rio de Janeiro, RJ, Brazil; ^2^Laboratory of Medicinal Plants and Derivatives, Rua Sizenando Nabuco 100, Manguinhos, 21041-250 Rio de Janeiro, RJ, Brazil; ^3^Pharmacy Faculty, Federal Fluminense University, R. Dr. Mario Vianna 523, Santa Rosa, 24241-002 Niterói, RJ, Brazil; ^4^FUNTAC, Av. das Acácias, Lote 01, Zona A, Distrito Industrial, 69917-100 Rio Branco, AC, Brazil; ^5^Chromatography Laboratory, Chemistry Department, Federal University of Amazonas, Av. Rodrigo Otávio 3000, Japiim, 69077-00 Manaus, AM, Brazil

## Abstract

Based on the ethnopharmacological evidences about the antileishmanial activity of* Copaifera* spp. oleoresins, the effects of crude extracts and fractions of oleoresin of two specimens from* Copaifera paupera* were evaluated on* Leishmania amazonensis* and* Leishmania infantum* strains. The oleoresin rich in *α*-copaene (38.8%) exhibited the best activity against* L. amazonensis* (IC_50_ = 62.5 *μ*g/mL) and against* L. infantum* (IC_50_ = 65.9 *μ*g/mL). The sesquiterpene *α*-copaene isolated was tested alone and exhibited high antileishmanial activity* in vitro* with IC_50_ values for* L. amazonensis* and* L. infantum* of 17.2 and 11.4 *μ*g/mL, respectively. In order to increase antileishmanial activity, nanoemulsions containing copaiba oleoresin and *α*-copaene were developed and assayed against* L. amazonensis* and* L. infantum* promastigotes. The nanoemulsion containing *α*-copaene (NANOCOPAEN) showed the best activity against both species, with IC_50_ of 2.5 and 2.2 *μ*g/mL, respectively. This is the first report about the antileishmanial activity of *α*-copaene.

## 1. Introduction

The* Copaifera* genus is characterized by shrubs or trees up to 40 meters high and sources of wood for the production of furniture and oleoresin (copaiba oil), extracted from its trunk, which has industrial applications. Species of* Copaifera* genus are mainly distributed in tropical America, with about 40 species, but are also present in tropical Africa with 4 species and in Asia, with 1 species,* Copaifera palustris* (Symington) De Wit, which is found in Malaysia [1, 2]. Popularly, the copaiba oil is used as anti-inflammatory and bactericidal. Some ethnopharmacological studies have suggested that* Copaifera* spp. oleoresins have antileishmanial activity [3–5].

Leishmaniasis is an endemic infectious disease in dozens of countries, including Brazil. The disease is caused by protozoa of the genus* Leishmania* (family Trypanosomatidae), whose vector is the female phlebotomine sandfly. There are three types of clinical manifestations: cutaneous, mucocutaneous, and visceral forms. The cutaneous form is the most common, frequently caused by* Leishmania major* and* Leishmania tropica* in Europe, Middle East, and Africa and by* Leishmania braziliensis* and* Leishmania mexicana* in Mexico, Central America, and South America [6]. Visceral leishmaniasis is frequently related to* Leishmania donovani *[7].

Conventional treatments with Glucantime, Pentostam, and Pentamidine are frequently ineffective [6]. Medicinal plants are important sources of new drugs and various studies have approached the incessant search for new therapeutic targets. Amaral et al. [8] described the effects of *α*-turmerone-rich hexane fraction from* Curcuma longa* against* L. amazonensis *promastigote forms. In that study, the entrapment of this hexane fraction into liposome lowered MIC values from 125 to 5.5 *μ*g/mL. Studies evaluating the antileishmanial activity of the labdane diterpenoid andrographolide and its nanoformulations were also reported. The free form of this diterpenoid was able to inhibit the intracellular amastigotes of* L. donovani* at IC_50_ values of 160 and 141 *μ*M, respectively. After andrographolide loading into nanoparticles (formulation prepared with 4% PVA), the antileishmanial activity was significantly improved when compared with the free drug (IC_50_ = 36 *μ*M) [9].

In literature, there is little information on the relationship between chemical composition of* Copaifera *spp. oleoresins and their biological activities. The chemical composition varies according to species of* Copaifera*, region of collection, and specimens, which makes it difficult to identify the substance responsible for the activity.* In vivo* assays employing mice infected with* Leishmania amazonensis* suggested that the oral treatment with* Copaifera martii* oleoresin causes reduction in the lesions, but the mechanism of action is still unclear [10]. Tincusi et al. [11] isolated sixteen compounds of the* C. paupera* oil and tested ten of them against* Leishmania tropica* promastigote forms. Despite the traditional use as antimicrobial, the authors verified that the pure substances [(−)-copalic acid, (−)-methyl copalate, agathic acid 15-methyl ester, (−)-polyalthic acid, (−)-methyl polyalthate, (−)-pinifolic acid, (−)-methyl-18-hydroxy-copaiferolate, (−)-kaurenoic acid, and 16*β*-kauran-19-oic acid] had no activity in the tests and concluded that it was still not possible to predict the antileishmanial activity of that copaiba oil.

The use of nanotechnology to develop formulations with applications in human health is getting popular among researchers looking for promising alternative for the treatment of various diseases. Regarding the use of products derived from plant raw materials, especially those containing essential oils in its composition, the nanoparticles are efficient in solving problems related to their physicochemical properties. They increase the stability of these oils when exposed to high temperatures, oxidation by atmospheric oxygen, and electromagnetic radiation, thus decreasing losses due to evaporation and decomposition of the active constituents. It is believed that the nanoemulsions (fine water in oil dispersions) have antimicrobial properties due to the small size of the oil particles with high surface tension. These characteristics can damage cell membranes of microorganisms causing a synergistic effect with the active components in the formulation and thus allowing the reduction of the content of substances that cause undesirable side effects [12].

Stable emulsions, especially where synthetic surfactants are used, are best formulated with emulsifiers or combination of emulsifiers having hydrophile-lipophile balance (HLB) values close to that required of the oil phase [13]. The HLB number is a semiempirical scale for selecting surfactants [14]. Because knowledge of the emulsification properties of different surfactants is of paramount importance in the industry of emulsions, the determination of these properties (such as HLB) in new possible emulsifiers is highly desirable [15]. In the same way, the determination of required HLB value of the oil phase appears as a critical step for development of emulsions and other semisolid formulations. In addition, the number of experiments can be reduced early during the formulation screening stage by using these parameters [16].

Nanoemulsions are dispersions of droplet size usually in the range 20–200 nm. It is obtained by the high energy emulsification methods, such as high-shear stirring, high-pressure homogenizers, and ultrasound generators. Due to their characteristic size, it possesses stability against sedimentation, creaming, flocculation, or coalescence. This long-term physical stability of nanoemulsions makes them unique, and they are sometimes referred to as “approaching thermodynamic stability” [17, 18]. Nanoformulations are being developed to improve the therapeutic arsenal on neglected diseases that affect populations in developing countries, such as leishmaniasis, malaria, and Chagas disease [19–24]. Formulations based on nanotechnology can increase the antileishmanial activity of flavonoid [25], diterpene [26, 27], saponine [28], and secoiridoid derivatives [29]. Physicochemical parameters for nanoemulsions containing crude* Copaifera multijuga* oleoresins with high content of *β*-caryophyllene were developed [30].

In this context, the aim of this work was to develop nanoemulsions containing* Copaifera paupera* (Herzog) Dwyer oleoresin crude extract, fraction, and its main compound. Additionally, the activity of these crude extracts, fraction, main compound, and their formulations was tested on* L. amazonensis* and* L. infantum* promastigote forms.

## 2. Material and Methods

### 2.1. Plant Material

The copaiba oleoresins (RE04 and RB13) of two specimens (trees E04 and B13) from Reserva Chico Mendes, Acre State/Brazil, were identified as* Copaifera paupera*, codified as E04 and B13. Voucher specimen was deposited at the Herbarium of Goeldi Museum/Pará State (Number 35,524).

### 2.2. Chemicals

Sorbitan oleate (Span 80®) and Polysorbate 20 (Tween 20®) were purchased from La Belle Ativos Ltda (Paraná, Brazil). Kaurenoic acid was purchased from Sigma-Aldrich (USA) and *n*-alkane standard (C7–C30) from Supelco (Bellefonte, PA, USA).

### 2.3. Isolation of *α*-Copaene

Copaiba oleoresin RE04 was submitted to preparative thin-layer chromatograph for the isolation of *α*-copaene. Aliquots containing 40 mg of the oleoresin were applied to aluminum sheets covered by the stationary phase silica gel 60 and eluted with hexane/ethyl acetate (7 : 3). The sesquiterpene was compared to authentic sample and confirmed by spectrometric and spectroscopic methods. 8 mg of *α*-copaene was obtained.

### 2.4. Fraction Rich in Kaurene (DIT B13)

The crude copaiba oleoresin of B13 specimen (3 g) was heated in three cycles of 40 minutes in microwave oven at medium power with intervals of 20 min for 3 hours. This procedure furnished 2.4 g of a fraction rich in kaurene diterpene (DIT B13) identified by GC-MS.

### 2.5. Gas-Chromatography: Mass Spectrometry (GC-MS) Analysis

Samples were analyzed and identified by GC-MS on an Agilent 6890 N GC coupled to a quadrupolar mass spectrometer (Agilent 5973N), with ionization by electronic impact (70 eV). The apparatus was fitted with a DB-5MS column (30 m × 0.25 mm I.D., 0.25 *μ*m phase film). The injector temperature was 290°C, ion source was 230°C, and the scan-range was 40–700* m/z*. The column temperature varied from 70°C to 310°C, at a rate of 5°C/min. Helium was the carrier gas at initial flow rate of 0.5 mL/min. The injected volume was 1 *μ*L, in split rate of 10 : 1. Interpretation and identification of the fragmentation mass spectrum were carried out by comparison with the Wiley NBS mass spectrum database of the GC-MS equipment. The retention indices of constituents were calculated using the equation of van Den Dool and Kratz [31], normalized to the retention times of *n*-alkane homologous series and compared with literature [32]. Results were expressed as relative percentage of peak area in chromatogram.

### 2.6. Quantification by GC-FID Analysis

Samples were analyzed by gas chromatography (Agilent 6890N GC) equipped with a flame ionization detector (GC-FID). All analyses were performed in five replicates and injected volume was 1 *μ*L, in splitless injection. The apparatus was fitted with a DB-5 column (30 m × 0.25 mm ID, 0.25 *μ*m phase film). The injector temperature was 290°C and the column temperature varied from 70°C to 310°C, at a rate of 5°C/min. The detector temperature was 300°C. Nitrogen was the carrier gas at initial flow rate 0.5 mL/min. Quantifications were performed by external standardization. Standard curves were constructed with five levels of analysis, ranging from 0.02375 to 0.76 mg/mL of *α*-copaene diluted in methanol.

### 2.7. *Leishmania* Culture

*Leishmania amazonensis* (IFLA/BR/1967/PH8) and* L. infantum* (MHOM/BR/1974/PP75) promastigote forms were obtained from the* Leishmania* Type Culture Collection of Oswaldo Cruz Institute/Fiocruz (Rio de Janeiro/RJ/Brazil). The parasites were maintained by weekly transfers in PBHIL medium supplemented with 10% fetal bovine serum (FBS), at 26°C [33].

### 2.8. Antileishmanial Activity and Inhibitory Concentrations Evaluation

Promastigote forms of* L. amazonensis* and* L. infantum* were harvested after 120 h culture (early stationary phase), washed twice with phosphate buffer saline (150 mM NaCl; 20 mM phosphate buffer, PBS, pH 7.2), and resuspended at the concentration of 5.0 × 10^6^ parasites/mL. Then 100 *μ*L of the cellular suspension was added to 96-well microtiter plates containing different concentrations of copaiba oleoresins (RE04 and RB13), DITB13 fraction, *α*-copaene, or their nanoformulations, leading to final test concentrations ranging from 1 to 500 *μ*g/mL. The microtiter plates were incubated at 26°C for 120 hours in order to evaluate parasites growth inhibition. Negative controls for each sample were made by treating parasites with the highest concentration of DMSO or empty nanoemulsions used in this study. After the incubation period,* L. amazonensis* and* L. infantum* cultures were subjected to the microplate technique based on the reduction of resazurin as previously described [34]. The 50% inhibitory concentration (IC_50_) was calculated by regression analysis using Microsoft Excel 2013. Assays were performed in triplicate (*n* = 3) and the IC_50_ values were expressed as *μ*g/mL ± standard error. Statistical analysis of the differences between IC_50_ values obtained for oleoresin or fraction samples and their respective nanoformulations were evaluated by Student's *t*-test. The differences were considered statistically significant whether *p* < 0.05.

### 2.9. Nanoemulsions Production

#### 2.9.1. Evaluation of Required HLB Value from Copaiba Oleoresin

The required HLB value from copaiba oil was determined as described by Fernandes et al. [35]. Emulsions were prepared at a final mass of 25 g, containing 85% (w/w) of distilled water, 10% (w/w) of copaiba oil, and the emulsifiers (Tween 20 and Span 80) at total blend concentration of 5% (w/w). The required amounts of Tween 20 and Span 80 were dissolved in the oil phase. The oil and aqueous phases were heated to 75 ± 5°C, separately, and mixed by the inversion method with mechanical stirring (400 rpm) for 20 min. Series of emulsions with HLB values ranging from 4.3 to 16.7 were prepared by blending together the emulsifiers in different ratios. A second set of emulsions was later prepared using smaller ratio intervals between the two most stable emulsions from the first series.

#### 2.9.2. Preparation of Nanoemulsion

Four nanoemulsions were developed employing as the oil phase five different samples: two samples of oleoresin crude extract from the different specimens of* C. pauper*a (NANORE04 and NANORB13), a fraction rich in the diterpene kaurene from B13 (NANODIT B13), and *α*-copaene (NANOCOPAEN). Formulations were prepared at a final mass of 10 g, containing 96.25% (w/w) of distilled water, 2.5% (w/w) of oil, and Tween 20 and Span 80 (1 : 0.16) at total blend concentration of 1.25% (w/w). The emulsifiers were dissolved in the oil phase. The aqueous phase was added dropwise into the oil phase under ultrasonic homogenization (Omini Sonic Ruptor 250™, Omni Inc.) at 100 W for 6 min. A blank emulsion was prepared as described without loading the oil. Nanoemulsions obtained were stored at room temperature and characterized as the droplet size distribution and Zeta potential by dynamic light scattering (DLS) using a Zeta Potential Analyzer (ZetaPlus™, Brookhaven Inst. Corp.). Each emulsion was diluted using ultrapure Milli-Q water (1 : 25). The analyses were performed in quintuplicate. The average droplet size was expressed as the mean diameter. Following the particle size analyses of the samples, the electrode was placed inside the cuvette, and five measurements of the Zeta potential were recorded.

#### 2.9.3. Nanoemulsions Evaluation by Transmission Electron Microscopy (TEM)

The morphology of *α*-copaene-loaded nanoemulsion was observed using a FEI Morgagni F 268 (Eindhoven, The Netherlands) transmission electron microscope. Samples were previously diluted in distillated water (1 : 20), filtered through 0.45 *μ*m membrane, and placed on copper grids. The dried grids were scanned at an accelerating voltage of 80 kV.

## 3. Results and Discussion

Table 1 presents the list of compounds identified in the* C. paupera* oleoresin samples of E04 and B13. The main differences observed among the oleoresins samples were the concentrations of the sesquiterpenes *α*-copaene (21.8% in RB13 and 38.8% in RE04),* trans*-caryophyllene (4.1% in RB13 and 21.4% in RE04), *δ*-cadinene (7.7% in RE04), caryophyllene oxide (12.5% in RB13), and the diterpene kaurene (33.2% in RB13 and 2.4% in RE04).

In general, the chemical composition of the copaiba oleoresin is quite variable among species of* Copaifera* genus as well as among individuals of the same species. A great seasonal variation has been often reported, which can influence the pharmacological activity [36–39]. Sesquiterpenes and diterpenes predominate copaiba oils, and abundance of *β*-caryophyllene is often related [36, 37, 40–42]. The amount of *β*-caryophyllene in the oleoresins of* Copaifera* can vary from 0.7% to 62.6% in* C. reticulata* [37] and from 5% to 60% in* C. multijuga* [36]. The main sesquiterpenes content of the oleoresin collected of* C. paupera* specimen from Tarauacá (Acre, Brazil) were *β*-bisabolene (20.2%) and *α*-zingiberene (19.4%), and the main diterpenes were kaurenoic acid (13.3%) and copalic acid (6.1%) [41].

In our work, the *α*-copaene content was higher than 20%, a significant difference compared to the oleoresins of other* Copaifera* species whose concentrations were usually detected below 6% [37, 43–45]. Only a study with two oleoresins collected in Xapuri (Acre, Brazil) showed *α*-copaene as main constituent [38].

The diterpene kaurene may be present in the oil of* Copaifera *genus, but not so frequently. The substance can be found in the oils of root wood, root bark, trunk wood, and trunk bark of a specimen of* C. langsdorffii* collected in Ceará (Brazil) achieving 30.2% of the trunk wood oil [40]. Kaurene was also found in the oil obtained by hydrodistillation of some specimens of* C. langsdorffii* collected in Lavras (Minas Gerais State, Brazil). The highest content achieved was 4.4% of the seed oil [46]. Gelmini et al. [44] also detected this diterpene in the oleoresin from* Copaifera langsdorffii*. The content was 1.57% determined by GC-MS analyses. From these data it is possible to conclude that the high content of kaurene (33.2%) in the oleoresin obtained from B13 in this work differs from the great majority of Copaiba oils studied.

After the chemical characterization of RE04 and RB13 crude oleoresins,* in vitro* assays against* L. amazonensis* and* L. infantum *promastigotes were performed and the 50% inhibitory concentrations (IC_50_) were determined for each sample (Table 2). Among the tested crude extracts, the copaiba RE04 oleoresin, rich in *α*-copaene, showed the best activity against* L. amazonensis* (IC_50_ = 62.5 *μ*g/mL) and against* L. infantum* (IC_50_ = 65.9 *μ*g/mL). Santos et al. [41] tested the antileishmanial activity of eight oils from different* Copaifera* spp.:* C. reticulata *(*β*-caryophyllene, *α*-copaene, and bergamotene),* C. martii *(kovalenic acid, bisabolene),* C. cearensis *(*β*-caryophyllene, *α*-copaene),* C. paupera *(bisabolene, zingiberene),* C. langsdorffii *(kaurenoic acid, *β*-caryophyllene),* C. officinalis *(hardwickiic acid, copalic acid),* C. multijuga *(*β*-caryophyllene, copalic acid), and* C. lucens* (polyalthic acid, copalic acid). The authors showed that all oleoresin samples were active against* L. amazonensis* promastigotes, with IC_50_ varying from 5.0 *μ*g/mL (*C. reticulata* from Pará State, Brazil) to 22.0 *μ*g/mL (*C. reticulata* from Acre State, Brazil) [47].

Despite the evidences of the antileishmanial activity of* Copaifera* spp. oleoresins it seems that it may depend on the crude extract composition. The total sesquiterpene and diterpene contents varied from species and region of collection. This variation could explain the large differences found on the* Copaifera* species antileishmanial activity observed to the exudates in the present work with others reported in literature.

The main sesquiterpene of our oleoresin, *α*-copaene, was not tested before and demonstrated an antileishmanial activity with IC_50_ values for* L. amazonensis* and* L. infantum* of 17.2 and 11.4 *μ*g/mL, respectively. So, *α*-copaene is an important constituent of the copaiba oil with accentuated antileishmanial activity* in vitro*. As far as we know, this is the first report about the antileishmanial activity of *α*-copaene.

Diterpene acids (agathic acid, hydroxycopalic acid, kaurenoic acid, methyl copalate, pinifolic acid, and polyaltic acid) recently isolated from* Copaifera* oleoresins exhibited moderate to high activity against promastigote and amastigote forms of* L. amazonensis *[48]. Hydroxycopalic acid exhibited the highest activity against promastigotes, with IC_50_ of 2.5 *μ*g/mL. Kaurenoic acid and pinifolic acid showed IC_50_ of 3.5 *μ*g/mL and 4.0 *μ*g/mL, respectively. These findings evidenced that diterpenes of copaiba oils should be better investigated. During the preparation of this sample RB13 in the microwave it was observed that heating the resin for more than three hours produced a brown polymer material. Thus, the time of three hours was used to obtain the fraction enriched in kaurene. The GC-MS analysis of the resulting fraction rich in kaurene (DIT B13) obtained by this alternative process is shown in Table 1. After assays, DIT B13 exhibited IC_50_ of 167.5 and 176.0 *μ*g/mL against* L. amazonensis *and* L. infantum*, respectively (Table 2). It is worth mentioning that this sample is rich in kaurene (64.8%). Until now, there is no study about the possible antileishmanial activity of kaurene described in literature. Overall, the sesquiterpenes found in the oleoresin, efficiently reduced by heating on microwave oven, can be associated as the most important constituents for the antileishmanial activity of copaiba oleoresins.

Contradictory antileishmaniasis activities have been attributed to the sesquiterpene *β*-caryophyllene. In a previous work, *β*-caryophyllene was tested against the promastigote and amastigote forms of* L. amazonensis* (MHOM/BR/77LTB0016 strain) and exhibited some activity only against the promastigote form, with IC_50_ of 96 *μ*M [49]. However, Soares et al. [39] reported activity against intracellular amastigotes of* L. amazonensis* (MHOM/BR/75/Josefa strain), with IC_50_ of 1.3 *μ*g/mL (6.4 *μ*M). According to literature [49, 50], another sesquiterpene also found in the oil of B13, *β*-caryophyllene oxide, was not active against* L. amazonensis*.

Thus, the importance of leishmaniasis disease and the results obtained with the copaiba oleoresin samples motivated the search for increasing the antileishmanial activity of the samples studied using nanoemulsion formulations. Several drug delivery systems have been proposed to combat leishmaniasis. In fact, different colloidal carriers such as liposomes, niosomes, and nanoemulsions are able to deliver drugs from natural or synthetic origin to specific cellular targets. The antileishmanial activity of different phytochemicals successfully incorporated to drug delivery systems was recently reviewed [20].

First, optimized process parameters found in the preliminary study were applied as the heat temperature of the oil phase and the choice of the surfactant couple (data not shown). Different surfactants were previously tested in order to select the best couple. In this sense, the nonionic emulsifiers from fatty acid esters, Tween 20 and Span 80, showed the best results and were used at total blend of 5% w/w at different ratios to evaluate the required HLB value from copaiba oil according to the literature [35]. Stable emulsions such as nanoemulsions, especially where synthetic surfactants are used, are best formulated with emulsifiers or combination of emulsifiers having HLB (hydrophile–lipophile balance) values close to that required of the oil phase [13]. The HLB number is a semiempirical scale for selecting surfactants [14] and the determination of required HLB value of the oil phase appears as a critical step for development of emulsions and other semisolid formulations. In addition, the number of experiments can be reduced early during the formulation screening stage by using these parameters [16].

The emulsions at the lower extreme of HLB range (4.3 to 9.0) cracked right after manipulation. The emulsion formulated with HLB 15.0 was finer and showed some bluish reflection to the naked eye, known as Tyndall effect, which is characteristic for nanoemulsions. The microscopic analysis (100x) corroborates with this observation. According to the fact that minimum droplet diameter is related to the required HLB and emulsion stability, it is proposed that the most stable emulsion is the one which was formulated with the HLB of surfactants mixture nearest to required HLB of the oil phase [51, 52]. In this sense, we evaluated all emulsions until 2 months. After this period of storage, the emulsion with HLB value 15.0 showed no micro or macroscopic changes. It maintained its fine appearance and bluish reflection observed after the manipulation.

**Figure 1:**
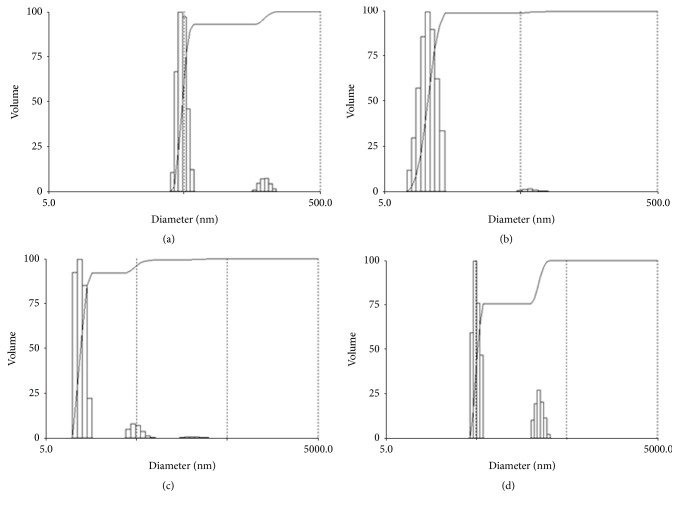
Droplet size distribution for nanoemulsions (a) NANORE04, (b) NANORB13, (c) NANODIT B13, and (d) NANOCOPAEN.

**Figure 2:**
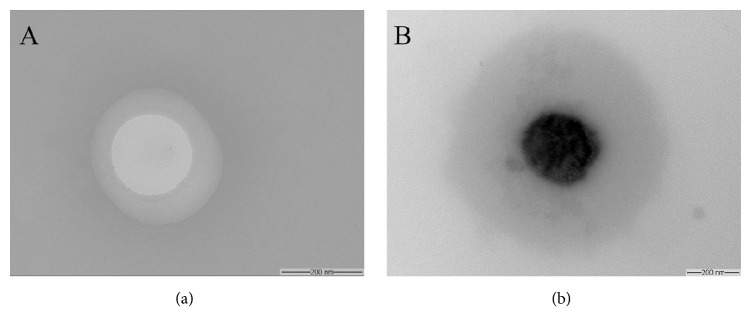
Photomicrographs of empty (a) and copaiba oleoresin-loaded (b) nanoemulsions.

Based on this results, the nanoemulsions were formulated using the ratio of surfactants (Tween 20: Span 80, 1 : 0.16) equal to the required HLB value for the copaiba oil (15.0). Intending to avoid the heat of the oil phase, the homogenization step was realized employing high energy input by ultrasound homogenization. Ultrasonic emulsification is considered very efficient in reducing droplet size, especially for small batches [18]. The emulsions developed with both copaiba oleoresins (RE04 and RB13), the fraction rich in kaurene (DIT B13), and the sesquiterpene *α*-copaene employed as oil phase showed mean diameter in the range 24.5–200 nm and low polydispersity (PDI). Thus, all formulations were characterized as nanoemulsions according to Solans et al. [18]. The size distribution for all nanoemulsions is shown in Figure 1. The nanoemulsion of RB13 (NANORB13) showed the smaller droplet size. The Zeta potential showed no significant difference between the analyzed samples. The characterization results are summarized in Table 3.  Figure 2 shows TEM image of NANOCOPAEN that confirmed the spherical morphology and size around 200 nm in diameter, besides the encapsulation of the bioactive sesquiterpene.

Dias et al. [30] also developed nanoemulsions containing copaiba oleoresin. In their work the formulation developed with the* C. multijuga* oleoresin in MTC, Tween 20, and Span 80, based on nanoemulsions using high-pressure homogenization, showed advantages over the others since it contained higher *β*-caryophyllene content and physical stability. In this context, the evaluated storage temperature (4°C) prevented flocculation and lower losses of volatile constituents.

In our study, nanoemulsions prepared with the crude oleoresins of* C. paupera* (NANORB13, NANORE04) and *α*-copaene (NANOCOPAEN) were assayed against* L. amazonensis *and* L. infantum *(Table 4). As expected, the nanoemulsions were able to enhance significantly the antileishmanial activity of those substances in all systems tested (*p* < 0.05). The nanoemulsion containing *α*-copaene (NANOCOPAEN) showed the best activity, with IC_50_ of 2.5 and 2.2 *μ*g/mL against* L. amazonensis* and* L. infantum* promastigote forms, respectively. Among the oleoresins, the best activity was observed with the nanoemulsion containing RE04 (NANORE04), rich in *α*-copaene, which showed IC_50_ of 17.3 and 39.8 *μ*g/mL against* L. amazonensis* and* L. infantum*, respectively. The formulation also improved the activity of DIT B13 (NANODIT B13), which exhibited IC_50_ of 28.9 and 54.7 *μ*g/mL against* L. amazonensis* and* L. infantum*, respectively (Table 4).

As *α*-copaene is abundant in all samples tested of oleoresin and demonstrated better antileishmanial activity than the crude exudates, its content in the nanoformulations was determined by GC-FID analyses. The results showed NANORE04 and NANORB13 with 0.33 and 0.13 mg/mL of *α*-copaene, respectively. After heating in microwave oven, *α*-copaene was detected at 0.04 mg/mL in the formulated kaurene-rich fraction NANODIT B13. The concentration of *α*-copaene incorporated into NANOCOPAEN was 1.81 mg/mL, which represents 7% of incorporation into nanoemulsion. These results are consistent with the antileishmanial activity observed in this study and with the different content of *α*-copaene in RE04 and RB13 oleoresins.

Taken together, these results further support the use of phytocompound nanoformulations as a promising approach for antileishmanial drugs discovery.

## 4. Conclusions

In conclusion, the results presented herein evidenced the* in vitro* antileishmanial activity of copaiba oleoresins. The sample RE04 demonstrated to be the most active oleoresin against* L. amazonensis* and* L. infantum *promastigotes and *α*-copaene showed better activity than the crude exudates. Additionally, RE04 and *α*-copaene nanoemulsions significantly increased the antileishmanial activity of these samples. This study provides contributions of new insights about the development and use of copaene as nanoemulsion and antileishmanial agent.

## Figures and Tables

**Table 1:** GC-MS analyses of oleoresins RB13, RE04, and DIT B13 fraction.

Substances	RI^lit.^ [32]	RI^cal.^	RB13 (%)	RE04 (%)	DIT B13 (%)
*α*-Cubebene	1351	1352	3.5	2.0	0.8
*α*-Copaene	1374	1378	21.8	38.8	5.7
*β*-Cubebene	1390	1389	4.4	10.1	-
*trans*-Caryophyllene	1417	1423	4.1	21.4	-
*α*-Bergamotene	1432	1434	2.8	1.1	0.6
Aromadendrene	1439	1441	1.3	2.8	0.4
*δ*-Selinene	1475	1473	-	-	1.7
Germacrene D	1480	1483	-	5.3	-
*β*-Selinene	1489	1492	4.0	2.5	1.0
Bicyclogermacrene	1494	1495	-	2.5	-
*α*-Selinene	1498	1499	1.6	-	-
*β*-Bisabolene	1505	1509	2.1	2.2	-
*δ*–Cadinene	1522	1520	-	7.7	-
Caryophyllene oxide	1582	1587	12.5	-	5.4
Fonenol	1590	1592	1.7	-	2.2
Caryophylla-3,8(13)-dien-5*β*-ol	1656	1660	-	-	1.7
14-Hydroxy-*α*-muurolene	1775	1779	2.3	-	1.0
Kaurene	2034	2036	33.2	2.4	64.8
Kaurenoic acid	-	-		1.1	-

Total sesquiterpenes			62.1	93.9	20.5
Total diterpenes			33.2	3.5	65.7
Total identified substances			95.3	97.4	85.3

**Table 2:** *In vitro* activity of the oleoresins (RB13 and RE04), fraction rich in kaurene (DIT B13), and *α*-copaene against *L. amazonensis* and *L. infantum*^*∗*^.

	*RB13* IC_50_ (*μ*g/mL)	*RE04* IC_50_ (*μ*g/mL)	*DIT B13* IC_50_ (*μ*g/mL)	*α-Copaene* IC_50_ (*μ*g/mL)
*L. amazonensis*	104.5 ± 5.7	62.5 ± 8.3	167.5 ± 7.3	17.2 ± 3.1
*L. infantum*	202.9 ± 17.5	65.9 ± 8.8	176.0 ± 4.5	11.4 ± 1.3

^*∗*^IC_50_ expressed as *μ*g/mL ± standard error, *n* = 3.

**Table 3:** Characterization of nanoemulsions^*∗*^.

Samples	Droplet size (nm)	PDI	Zeta potential
NANORE04	114.9 ± 1.2	0.270	−24.46 ± 1.15
NANORB13	24.5 ± 0.3	0.294	−23.04 ± 1.90
NANODIT B13	112.6 ± 2.3	0.318	−23.96 ± 1.44
NANOCOPAEN	200.0 ± 2.3	0.192	−26.04 ± 2.25

^*∗*^Mean ± standard error (*n* = 5).

**Table 4:** *In vitro* activity of the nanoemulsions containing oleoresins (NANORE04, NANORB13), fraction rich in kaurene (NANODITB13), and *α*-copaene (NANOCOPAEN) against *L. amazonensis* and *L. infantum*^*∗*^.

	*Blank* (empty nano)	*NANORE04* IC_50_ (*μ*g/mL)	*NANORB13* IC_50_ (*μ*g/mL)	*NANODITB13* IC_50_ (*μ*g/mL)	*NANOCOPAEN* IC_50_ (*μ*g/mL)
*L. amazonensis*	n.a.	17.3 ± 2.1	34.7 ± 10.4	28.9 ± 3.7	2.5 ± 1.2
*L. infantum*	n.a.	39.8 ± 0.3	46.8 ± 8.8	54.7 ± 6.0	2.2 ± 0.8

^*∗*^IC_50_ expressed as *μ*g/mL ± standard error, *n* = 3; n.a.: not active.
